# Pyramiding of three bacterial blight resistance genes for broad-spectrum resistance in deepwater rice variety, Jalmagna

**DOI:** 10.1186/s12284-015-0051-8

**Published:** 2015-05-31

**Authors:** Sharat Kumar Pradhan, Deepak Kumar Nayak, Soumya Mohanty, Lambodar Behera, Saumya Ranjan Barik, Elssa Pandit, Srikanta Lenka, Annamalai Anandan

**Affiliations:** Crop Improvement Division, Central Rice Research Institute, Cuttack, Odisha 753006 India

**Keywords:** Deepwater rice, Marker-assisted selection, Bacterial blight gene pyramiding, Broad-spectrum resistance, Foreground selection, Background selection, *xa5*, *xa13*, *Xa21*

## Abstract

**Background:**

Jalmagna is a popular deepwater rice variety with farmers of India because of its good yield under waterlogged condition. However, the variety is highly susceptible to bacterial blight (BB) disease. The development of resistant cultivars has been the most effective and economical strategy to control the disease under deepwater situation. Three resistance genes (*xa5* + *xa13* + *Xa21*) were transferred from Swarna BB pyramid line, using a marker-assisted backcrossing (MAB) breeding strategy, into the BB-susceptible elite deepwater cultivar, Jalmagna.

**Results:**

Molecular marker integrated backcross breeding program has been employed to transfer three major BB resistance genes (*Xa21*, *xa13* and *xa5*) into Jalmagna variety. During backcross generations, markers closely linked to the three genes were used to select plants possessing these resistance genes and markers polymorphic between donor and recurrent parent were used to select plants that have maximum contribution from the recurrent parent genome. A selected BC_3_F_1_ plant was selfed to generate homozygous BC_3_F_2_ plants with different combinations of BB resistance genes. The three-gene pyramid and two gene pyramid lines exhibited high levels of resistance against the BB pathogen. Under conditions of BB infection, the three-gene pyramided lines exhibited a significant yield advantage over Jalmagna. The selected pyramided lines showed all agro-morphologic traits of Jalmagna without compromising the yield.

**Conclusion:**

The three major BB resistance genes pyramided lines exhibited high level of resistance and are expected to provide durable resistance under deep water situation where control through chemicals is less effective. High similarity in agro-morphologic traits and absence of antagonistic effects for yield and other characters were observed in the best pyramided lines.

## Background

Rice (*Oryza sativa* L.) is an important food crop that serves as a major carbohydrate source for nearly half of the world’s population. In India, it is grown in 43 million hectares accounting for 42% of food grain production and 55% of cereal production. To sustain self-sufficiency and to meet food grain requirement of future, India has to produce 135–140 million tones of rice by 2030. This has to necessarily meet from less land, less water, less labor and fewer chemicals, constant battle against new emerging pathogens and pests and possible adverse effects from climate change (Khush 2005). This ecosystem covers around 4 million hectares of which 3 million hectares are under deepwater ecology and 1 million hectare under very deepwater ecology (floating rice).Under deepwater ecology, the crop remains waterlogged for a period of more than a month with more than 50 cm water depth while in floating type the water depth remains more than one meter. The average productivity of deepwater ecosystem is around 1 t/ha while floating type is again very low yield. Bacterial leaf blight (BB) caused by *Xanthomonas oryza*e pv. *oryzae* (*Xoo*) is the most important disease of deepwater rice in India. In some areas of Asia, it can reduce crop yield by up to 50% (Khush et al. 1989) or even up to 80% (Singh et al. 1977). It also causes poor quality fodder. This affects photosynthetic areas and reduces the yield drastically and produce partial grain filling and low quality fodder yield.

Although a large number of rice varieties have been released for different agro-ecosystems in India, only a few are widely grown in deep water situations. Rice varieties, Jalmagna and Dinesh are very widely grown in deep water areas of India. Theses varieties are popular among rice farmers and consumers because of its high yield, medium slender grains and excellent cooking and eating qualities. Despite popularity, these varieties are highly susceptible to many pests and diseases including BB. BB is the most important disease of deepwater rice in India and has become a major production constraint. In absence of effective chemical or other control agents against the pathogen in deepwater situation, host plant resistance has gained enormous importance in controlling this disease (Devadath 1989). Therefore, host plant resistance offers the most effective, economical and environmentally safe option for management of BB pathogen in deepwater situation (Khush et al. 1989). In other ecology also, development of resistant cultivars carrying resistant genes have been the most effective and economical strategy to control BB disease and no environmental pollutions (Huang et al. 1997; Jena and MacKill 2008; Singh et al. 2001; Sundaram et al. 2008; Rajpurohit et al. 2011; Dokku et al. 2013; Suh et al. 2013). Globally, thirty eight BB resistance genes have been identified from diverse sources (Bhasin et al. 2012). A number of these resistance genes have been tagged by closely linked molecular markers (Yoshimura et al. 1995; Sonti 1998; Rao et al. 2002; Gu et al. 2008). A few of these genes like *Xa4* have been incorporated widely in many high yielding varieties through conventional breeding (Khush et al. 1989). However, widespread cultivation of varieties with *Xa4* has led to predominance of *Xoo* races that can overcome this gene (Mew et al. 1992). The deployment of rice cultivars that have multiple BB resistance genes is expected to lead to more durable resistance.

Pyramiding multiple R genes in a single line confers wide-spectrum and durable resistance. Tightly linked DNA markers have been developed for several BB resistance genes. The BB resistance genes, *Xa1, xa5, xa13, Xa21, Xa26* and *Xa27* have been cloned and used for breeding program. With the exception of *xa5* and *xa13*, the BB resistance genes are dominant in nature and the markers developed from the sequencing information of these genes are widely used in MAS (Song et al. 1995; Yoshimura et al. 1998; Gu et al. 2005; Chu et al. 2006a). Using the gene pyramid approach, improved *indica* rice cultivars with broad spectrum durable BB resistance have been developed by combining different genes (Huang et al. 1997; Sanchez et al. 2000; Shanti et al. 2001; Singh et al. 2001; Joseph et al. 2004; Pha and Lang 2004; Bharatkumar et al. 2008; Hu et al. 2008; Perez et al. 2008; Sundaram et al. 2008; Rajpurohit et al. 2011; Dokku et al. 2013; Suh et al. 2013). A three-gene combination appeared to be the most effective; with *Xa21* contributing the largest component of resistance. Therefore, incorporation of three BB resistant genes combination was taken up in the popular variety Jalmagna background by integrating marker-assisted backcrossing with phenotypic selection for development of pyramiding lines for the handicapped ecology.

## Results

### Pyramiding of bacterial blight resistance genes

The parent polymorphism was detected for the donor (CRMAS 2232–85) and recurrent parent (Jalmagna) with the markers pTA 248, RG 136 and xa5S, R (multiplex) for the genes *Xa 21*, *xa13* and *xa 5* respectively (Table 1). The parents were polymorphic with respect to these genes. In addition, the parents were screened with 236 rice microsatellite markers (Table 2) of which 120 were polymorphic and 60 were used for background selection.

**Table 1:** **Markers used for foreground selection of three bacterial blight resistance genes in marker-assisted backcross breeding**

**Resistance gene**	**Chromosome number**	**Marker**	**Primer sequences used for gene detection**		**Expected size (bp)**	**Band type**	**reference**
			Forward(5’-3’)	Reverse(5’-3’)			
*xa5*	5	xa5S (Multiplex)	GTCTGGAATTTGCTCGCGTTCG	TGGTAAAGTAGATACCTTATCAAACTGGA	410 bp, 310 bp,180 bp	STS	Sundaram et al. 2011
		xa5SR/R (Multiplex)	AGCTCGCCATTCAAGTTCTTGAG	TGACTTGGTTCTCCAAGGCTT			
*xa13*	8	RG136	TCCCAGAAAGCTACTACAGC	GCAGACTCCAGTTTGACTTC	530 bp, 490 bp	STS	Huang et al. 1997
*Xa21*	11	pTA248	AGACGCGGAAGGGTGGTTCCCGGA	AGACGCGGTAATCGAAGATGAAA	1000 bp	STS	Huang et al. 1997

**Table 2:** **Microsatellite markers those are polymorphic between Jalmagna and CRMAS 2232-85**

**Chromosome**	**No. of markers analyzed**	**Total no. of polymorphic markers**	**Name of the polymorphic markers**
1	25	11	RM23, RM48, RM212, RM272, RM575, RM428, RM488, SSR09, SSR 31, SSR 60 ,SSR 71
2	25	12	RM154, RM211, RM233A, RM263, RM475, RM45, RM530,SSR11, SSR 14, SSR 44 ,SSR 71 ,SSR 85
3	21	10	RM16, RM130, RM218, RM203,SSR 06, SSR 13, SSR 18, SSR 45, SSR 85, SSR 93
4	18	12	RM241, RM307, RM401, RM55, RM471, RM518, RM470, SSR 04, SSR 10, SSR 19, SSR 32, SSR 40
5	19	12	RM164, RM592, RM440, SSR 05, SSR 13, SSR 21, SSR 27, SSR 34,SSR 37, SSR 43, SSR 50, SSR 59
6	20	10	RM225, RM276, RM340, RM402, RM586, RM589, RM588, SSR 21, SSR 31, SSR 54
7	21	9	RM10, RM336, RM560, RM432, RM346, SSR 28, SSR 37, SSR 41, SSR 44
8	20	10	RM223, RM241, RM407, RM3395, RM6208, RM22550, RM22506, RM8271, SSR 14 ,SSR 48
9	16	7	RM219, RM242, RM257, RM410, RM3555,SSR 40, SSR 42
10	17	9	RM171, RM216, RM333, RM330, SSR 03, SSR 06, SSR 11, SSR 25, SSR 30
11	18	11	RM21, RM144, M202, RM206, RM209, RM260, RM287, SSR 3, SSR 4, SSR 11, SSR 27
12	17	7	RM17, RM195, RM415, RM23, SSR 23, SSR 26, SSR 36
Total	236	120	

**Figure 1:**
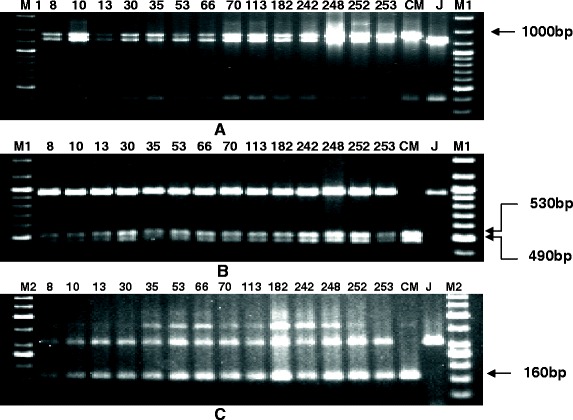
PCR amplification of markers linked to resistance genes *Xa21*, *xa13* and *xa5* using primers **A**) pAT248, **B**) RG136 and **C**) xa5S and xa5R of BC_1_F_1_. Lanes on the top of the gel shows the BC_1_F_1_ plant no., CM- CRMAS 2232-85, J-Jalmagna, M1-Molecular weight marker (100bp plus ladder), M2-Molecular weight marker (50bp ladder), Arrows indicate the resistance specific markers.

**Figure 2:**
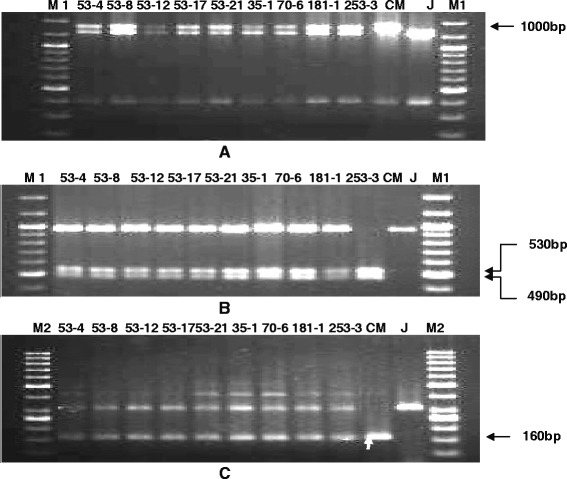
PCR amplification of markers linked to resistance genes *Xa21*, *xa13* and *xa5* using primers **A**) pAT248, **B**) RG136 and **C**) xa5S and xa5R of BC_2_F_1_. Lanes on the top of the gel shows the BC_2_F_1_ plant no., CM- CRMAS 2232-85, J-Jalmagna, M1-Molecular weight marker (100bp plus ladder), M2-Molecular weight marker (50bp ladder).

**Figure 3:**
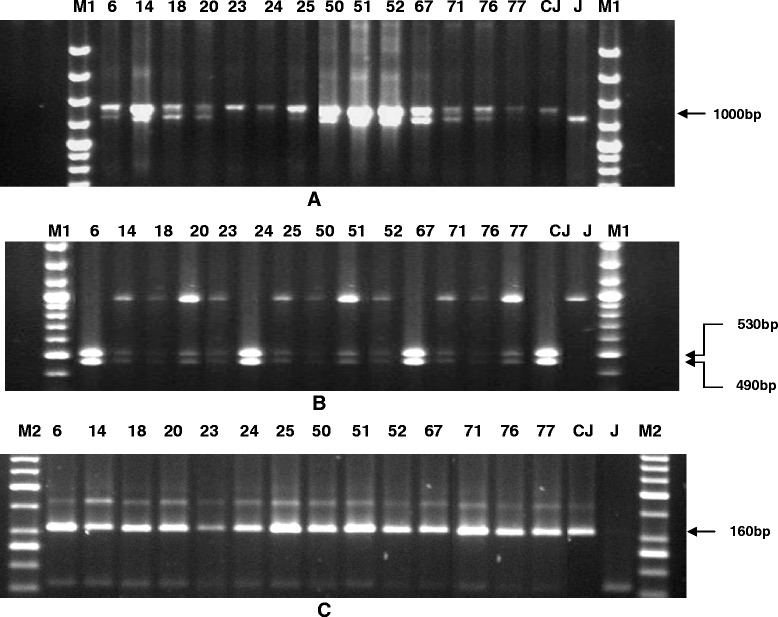
PCR amplification of markers linked to resistance genes, *Xa21*, *xa13* and *xa5* using primers **A**) pAT248, **B**) RG136 and **C**) xa5S and xa5R of BC_3_F_1_ plants. Lanes on the top of the gel shows the BC_3_F_1_ plant no. CM- CRMAS 2232-85, J-Jalmagna, M1-Molecular weight marker (100bp plus ladder), M2-Molecular weight marker (50bp ladder).

Molecular markers were integrated in the backcross breeding programme upto BC_3_F_2_ generation. During the breeding procedure, foreground selection was practiced from F_1_ generation till BC_3_F_3_ generation at each stage to select the plants having resistance alleles of the three target genes and only progenies having the resistance alleles were advanced for the next generation (Figures 1, 2 and 3). Background selection was started from BC_1_F_1_ to BC_3_F_1_ generation and in each step genotype possessing highest genome content of the recipient parent was selected to hybridize for next backcross. A total of 650 F_1_ plants were produced and 150 F_1_ plants were tested for the hybridity and confirmed by their heterozygosity for the resistance gene linked markers of which 143 plants were observed to be true F_1_s. The true F_1_s were backcrossed using Jalmagna as a recurrent parent. These crossed seeds were raised (360 BC_1_F_1_ seeds) for further backcrossing with Jalmagna. Ninety three BC_1_F_1_ plants showed the presence of *Xa21* resistance gene specific bands (1000 bp) while 91 plants showed the presence of *xa13* resistance gene specific bands (490 bp and 530 bp). One hundred sixteen BC_1_F_1_ plants showed the presence of *xa5* resistance gene specific bands (160 bp). Based on the amplification of resistance specific bands, 31 BC_1_F_1_ plants showed the presence of *Xa21* and *xa13* resistance genes while 42 BC_1_F_1_ plants showed the presence of *Xa21* and *xa5* resistance genes. Forty six BC_1_F_1_ plants showed the presence of *xa13* and *xa5* resistance genes. Only fourteen plants showed the presence of three BB resistance genes *Xa21, xa13* and *xa5*. Out of these 14 BC_1_F_1_ progenies, plant showing 77.5% of recurrent genome (Plant No.53) was backcrossed with recurrent parent Jalmagna (Table 3).

**Table 3:** **Number of triple resistant gene heterozygotes identified and estimation of recurrent parent genome contribution**

**Generation**	**# of plants** **scored** ^**a**^	**# of plants that are ** **triple heterozygotes** ^**a, b**^	**Estimated maximum % ** **contribution of recurrent ** **parent genome to selected ** **backcross plant** ^**c**^	**Expected % contribution ** **of recurrent parent genome** ** to selected backcross plant** ^**d**^
BC_1_F_1_	360	14	77.5	77.5
BC_2_F_1_	122	9	91.8	91.8
BC_3_F_1_	285	14	97	97

^a^At each backcross generation, genomic DNA was isolated from derivative lines and genotyping was performed using primers that are tightly linked to BB resistant genes as described in Materials and methods.

^b^At each backcross generation, fewer than expected triple heterozygotes were obtained. This is due to the fact that some of the

putative backcross progeny were obtained by inadvertent selfing of Jalmagna (the female parent in these backcrosses).

^c^At each backcross generation, genomic DNA was isolated from derivative lines that are triple heterozygotes for BB resistant gene linked markers. Microsatellite markers that are polymorphic between the parental lines were then used, as described in Materials and Methods, to identify the plant with maximum recurrent parent genome contribution.

^d^As per Mendelian ratios for independent gene action.

Total of 122 BC_2_F_1_ progenies were produced of which, twenty one, thirty three and thirty six BC_2_F_1_ plants showed presence of resistance genes, *Xa21, xa13* and *xa5*, respectively. Based on the amplification pattern, 11 BC_2_F_1_ plants showed the presence of *Xa21* and *xa13* resistance genes while 13 BC_2_F_1_ plants showed the presence of *Xa21* and *xa5* resistance genes. Twenty three BC_2_F_1_ plants showed the presence of *xa13* and *xa5* resistance genes. Only nine plants exhibited the amplification of three resistance genes *Xa21, xa13* and *xa5*. The background selection of these nine BC_2_F_1_ plants with sixty polymorphic SSR markers exhibited the presence of 88.13 % to 91.82 % with an average of 90.95% of recurrent genome content. The plant containing 91.82% genome content of Jalmagna (Plant No.53-21) was used for backcrossing (Table 3).

A total of 285 BC_3_F_1_ backcross derivative progenies were produced by backcrossing the plant showing 91.82% recurrent genome with the recipient parent, Jalmagna. Twenty eight BC_3_F_1_ plants were positive for *Xa 21*, 35 for *xa 5* and 14 for *xa 13*. Eighteen BC_3_F_1_ plants showed the presence of *Xa21* and *xa13* resistance genes while 14 plants showed the presence of *Xa21* and *xa5* resistance genes and 14 plants showed the presence of *xa13* and *xa5* resistance genes. Only fourteen plants showed the presence of three resistance genes *Xa21, xa13* and *xa5*. These BC_3_F_1_ plants showed recurrent genome content of Jalmagna ranging from 91 to 97% with an average of 92.38% (Table 3). BC_3_F_1_ derivative SPJ53-21-77 and and SPJ53-21- 25 showed more than 95% genome content of recipient parent were self pollinated to obtain the derivatives of BC_3_F_2_ generation. In BC_3_F_2_ generation, plants homozygous for three and two bacterial blight resistance gene combinations were identified. It is observed that 26 plants containing *Xa21, xa13* and *xa5* genes*; 31 plants with Xa21* and *xa5; 31 plants with Xa21, xa13* and 30 with *xa13* and *xa5* amongst the BC_3_F_2_ derivatives. The plants with three and two genes were grown as BC_3_F_3_ lines.

### Bioassays

Bioassays conducted against eight isolates of *Xoo* confirmed the resistance and susceptible reaction of the donor (CRMAS 2232–85) and the recurrent (Jalmagna) respectively with the donor showing smaller range of average lesion lengths (2.1-2.8 cm) while on Jalmagna, the lesion lengths were longer (9.4-12.8 cm) (Table 4). The results indicated that the pyramided lines were better as compared to recurrent parent, Jalmagna with regard to bacterial leaf blight tolerance. Screening of the BC_3_F_3_ pyramided lines against *Xoo* isolates exhibited that all the pyramid lines were more effective in comparison to the recipient parent. The lesion lengths observed on the lines containing *Xa21* + *xa13* gene combination varied from 3.1 to 3.9 cm ; for Xa21 + xa5 combination, 3.5-4.8 cm ; for *xa5* + *xa13* combination 4.9 to 5.7 while 1.4 to 2.9 cm lesion length present in pyramided line containing *xa5* + *xa13* + *Xa21* combination. The individual values for the donor parent and recurrent parent are in the range of 1.7–3.3 and 9.0–13.3 cm respectively. Though all the gene combinations tested did not show any susceptible reaction to any of the eight isolates employed, the gene pyramids with three genes displayed higher levels of disease resistance with shorter lesion lengths against all BB isolates. Results indicated that the degree of severity of the disease from the data, the order of gene combinations in conferring resistance was: *xa5* + *xa13* < *xa5* + *Xa21* < *xa13* + *Xa21* < *xa5* + *xa13* + *Xa21*.

**Table 4:** **Bacterial blight reaction of parental and BC**_**3**_ **F**_**3**_ **pyramided lines against different** ***Xoo*** **strains**

**Sl.** **No.**	**Pyramided lines/Xoo strains**	**Gene combination**	**Mean lesion length in cm (Mean ± standard error)**								
			**Xa-17**	**Xa-7**	**xa-2**	**xb-7**	**xc-4**	**xd-1**	**xa-1**	**xa-5**	**Disease reaction**
1	SPJ 53-21-06	*Xa21 + xa13 + xa5*	2.8 ± 0.4	2.5 ± 0.8	2.7 ± 0.9	1.9 ± 1.1	2.7 ± 1.0	2.6 ± 0.75	2.4 ± 0.56	2.5 ± 0.47	R
2	SPJ 53-21-14	*Xa21 + xa13 + xa5*	2.6 ± 0.6	2.7 ± 0.4	2.5 ± 1.1	2.5 ± 0.8	2.9 ± 0.7	2.8 ± 0.64	2.3 ± 0.43	2.3 ± 0.64	R
3	SPJ 53-21-18	*Xa21 + xa13 + xa5*	2.7 ± 0.3	2.9 ± 0.5	2.8 ± 1.2	1.8 ± 0.9	2.1 ± 0.9	2.3 ± 0.37	1.9 ± 0.72	2.1 ± 0.75	R
4	SPJ 53-21- 20	*Xa21 + xa13 + xa5*	2.8 ± 0.3	2.5 ± 0.4	2.7 ± 0.7	1.6 ± 0.5	1.6 ± 1.2	1.9 ± 0.52	2.6 ± 0.54	1.7 ± 0.83	R
5	SPJ 53-21- 23	*Xa21 + xa13 + xa5*	2.7 ± 0.5	2.6 ± 0.6	2.6 ± 1.5	2.1 ± 0.7	1.8 ± 1.1	1.7 ± 0.93	2.8 ± 0.71	1.4 ± 0.85	R
6	SPJ 53-21-24	*Xa21 + xa13 + xa5*	2.9 ± 0.4	2.8 ± 0.7	2.8 ± 0.5	2.3 ± 0.4	1.9 ± 1.3	2.9 ± 0.33	2.5 ± 0.83	2.5 ± 0.52	R
7	SPJ 53-21-25	*Xa21 + xa13 + xa5*	2.9 ± 0.5	2.7 ± 0.9	2.9 ± 1.3	2.4 ± 0.6	2.8 ± 0.8	2.6 ± 0.84	2.4 ± 0.95	2.7 ± 0.44	R
8	SPJ 53-21-50	*Xa21 + xa13 + xa5*	2.8 ± 0.3	2.4 ± 0.5	2.7 ± 0.7	1.6 ± 1.1	2.6 ± 0.7	2.1 ± 0.72	1.7 ± 1.2	1.7 ± 0.92	R
9	SPJ 53-21-51	*Xa21 + xa13 + xa5*	2.9 ± 0.4	2.7 ± 0.4	2.5 ± 0.8	2.3 ± 0.7	2.1 ± 0.6	1.6 ± 0.94	2.9 ± 0.52	1.4 ± 1.1	R
1	SPJ 53-21-52	*Xa21 + xa13 + xa5*	2.7 ± 0.3	2.8 ± 0.7	2.8 ± 1.1	1.7 ± 0.8	2.9 ± 0.5	2.2 ± 0.88	2.9 ± 0.47	1.9 ± 1.2	R
11	SPJ 53-21-67	*Xa21 + xa13 + xa5*	2.5 ± 0.7	2.6 ± 0.9	2.6 ± 0.3	1.9 ± 0.9	2.8 ± 0.8	2.5 ± 1.12	1.8 ± 0.85	2.3 ± 0.53	R
12	SPJ 53-21-71	*Xa21 + xa13 + xa5*	2.6 ± 0.6	2.5 ± 0.5	2.5 ± 0.7	2.2 ± 1.0	2.8 ± 0.4	2.7 ± 0.85	2.3 ± 0.63	2.8 ± 0.44	R
13	SPJ 53-21-76	*Xa21 + xa13 + xa5*	2.8 ± 0.4	2.6 ± 0.7	2.7 ± 0.6	2.1 ± 0.6	2.9 ± 0.5	2.9 ± 0.67	2.6 ± 0.52	2.9 ± 0.52	R
14	SPJ 53-21-77	*Xa21 + xa13 + xa5*	2.9 ± 0.3	2.7 ± 0.9	2.8 ± 1.2	2.4 ± 0.5	2.8 ± 0.4	2.9 ± 0.69	2.8 ± 0.73	2.8 ± 0.55	R
15	SPJ 53-21-27	*Xa21 + xa13*	3.6 ± 0.4	3.4 ± 1.1	3.5 ± 1.1	3.1 ± 0.6	3.4 ± 0.7	3.7 ± 0.4	3.6 ± 1.2	3.7 ± 0.63	MR
16	SPJ 53-21-15	*Xa21 + xa13*	3.9 ± 0.5	3.8 ± 1.2	3.6 ± 1.0	3.7 ± 0.5	3.7 ± 0.9	3.8 ± 0.77	3.4 ± 1.1	3.85 ± 0.7	MR
17	SPJ 53-21-38	*Xa21 + xa5*	4.1 ± 0.5	3.7 ± 1.4	3.9 ± 0.7	3.5 ± 0.6	3.7 ± 1.2	4.3 ± 0.7	4.2 ± 0.4	3.9 ± 0.7	MR
18	SPJ 53-21-66	*Xa21 + xa5*	4.5 ± 0.8	3.9 ± 1.3	4.4 ± 0.6	4.8 ± 0.8	3.9 ± 0.9	4.3 ± 0.5	4.2 ± 0.63	4.1 ± 0.65	MR
19	SPJ 53-21-13	*xa13 + xa5*	5.6 ± 1.1	5.3 ± 0.8	5.1 ± 0.6	4.9 ± 0.7	5.5 ± 1.3	5.3 ± 0.49	5.7 ± 0.72	4.9 ± 0.53	MR
20	SPJ 53-21-36	*xa13 + xa5*	5.4 ± 0.7	5.1 ± 0.8	5.0 ± 0.7	5.2 ± 0.7	5.6 ± 0.9	5.1 ± 0.76	5.2 ± 0.84	5.3 ± 0.65	MR
21	CRMAS 2232-85	*Xa21 + xa13 + xa5*	2.8 ± 0.4	2.5 ± 0.3	2.4 ± 0.4	2.1 ± 0.4	2.8 ± 0.5	2.4 ± 0.4	2.6 ± 0.8	2.2 ± 0.6	R
22	Jalmagna	-	12.6 ± 1.7	11.4 ± 1.4	12.8 ± 1.5	9.4 ± 1.2	11.6 ± 1.6	9.8 ± 1.8	10.2 ± 1.7	11.6 ± 1.8	S

### Yield and agro-morphological traits of the pyramided lines

**Figure 4:**
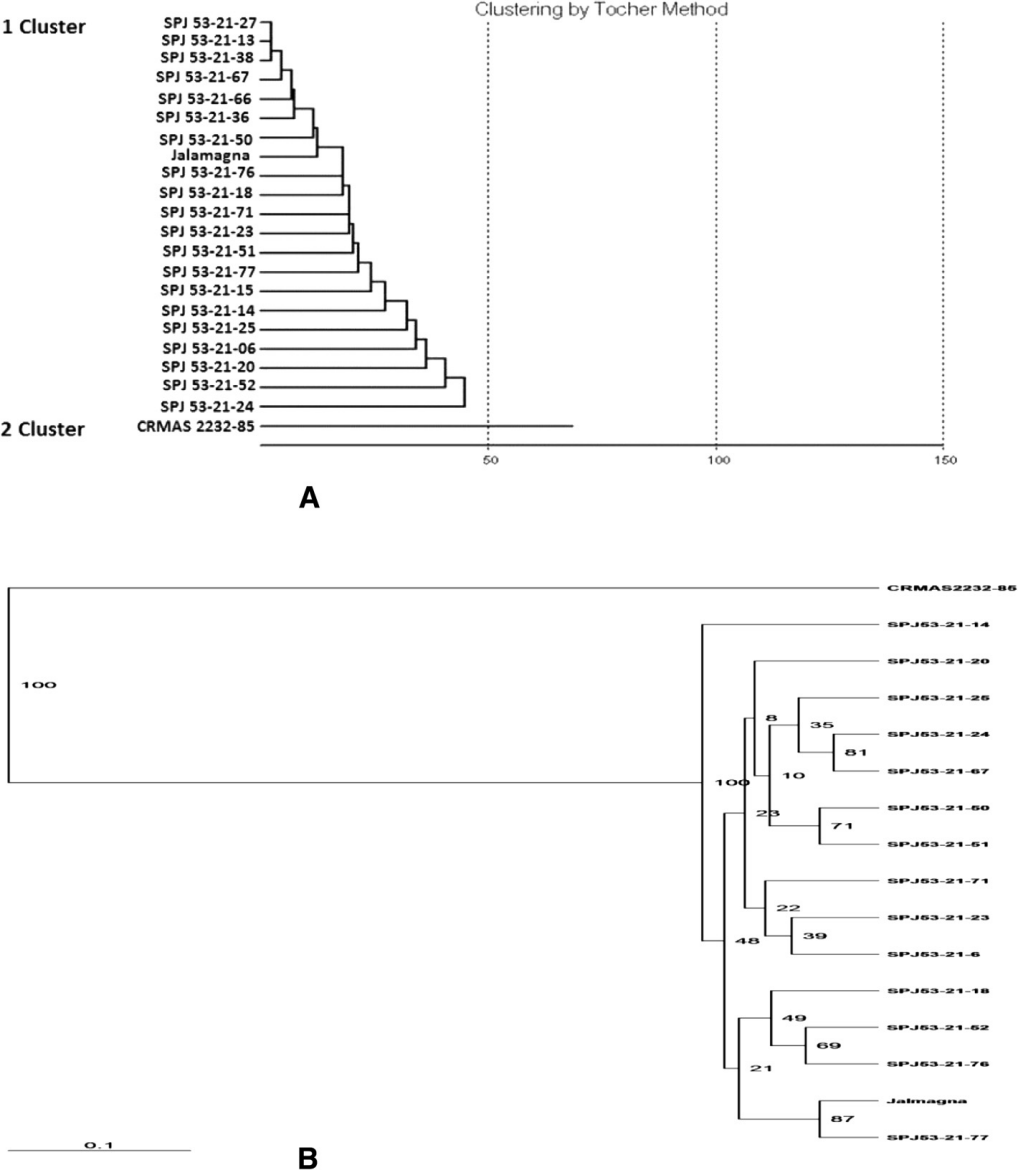
Dendrograms illustrating the genetic relationship between parents and pyramided lines (**A**) based on 14 agro-morphological traits; (**B**) based on microsatellite markers.

Fourteen three-gene pyramid and six two genes pyramid lines at BC_3_F_3_ generation along with the donor and recipient parents were evaluated during wet season, 2013 at CRRI, Cuttack. The recipient parent, Jalmagna recorded mean grain yield of 17.35 g/plant, while the donor parent (Swarna BB pyramided line) recorded 20.5 g/plant. The test entries *viz*., SPJ23, SPJ25, SPJ50, SPJ51, SPJ52 and SPJ77 showed grain yields higher than recurrent parent, Jalmagna (Table 5). Many test entries did not show any significant variation as compared to Jalmagna in terms of flowering duration, panicles/m^2^, plant stature as well as other characters that are considered under distinctness, uniformity and stability (DUS) tests. The genetic distance coefficient on 14 agro-morphologic traits of 20 pyramids and two parental lines revealed that two clusters were observed and it is interesting to note that all the pyramided lines are similar to the recipient parent, Jalmagna and are clubbed in cluster1 while in cluster 2, only solitary line the donor parent is accommodated. (Table 5; Figure 4A).

**Table 5:** **Agro-morphologic traits of pyramided and parental lines in BC**_**3**_ **F**_**3**_ **generation**

**Pyramided lines**	**Plant height (cm)**	**Days to 50% flow**	**Panicles/plant**	**Panicle length(cm)**	**No of grains/ panicle**	**Fertility %**	**1000- seed weight(g)**	**Single plant yield(g)**	**Grain length (mm)**	**Grain Breadth (mm)**	**Flag leaf length(cm)**	**Flag leaf breadth (cm)**	**2** ^**nd**^ **leaf length (cm)**	**2** ^**nd**^ **leaf breadth (cm)**	**Auricle colour**	**Collar colour**
SPJ 53-21-06	150	122	10	21.95	211.5	87	22.45	16.15	0.76	0.88	36.0	1.2	69.0	1.2	Green	yellowish
SPJ 53-21-14	178	124	12	22.1	204	86	22.05	16.85	0.73	0.875	39.0	1.2	49.0	1.2	Green	yellowish
SPJ 53-21-18	163.5	121	11	26.25	205.5	84	21.6	17	0.67	0.89	41.0	0.9	59.0	0.9	Green	yellowish
SPJ 53-21- 20	172.5	124	11	26.25	208	84	22.7	17.1	0.75	0.86	47.0	1.2	75.0	1.2	Green	yellowish
SPJ 53-21- 23	158.5	123	12	24.2	207.5	83	21.5	16.5	0.74	0.88	45.0	1.1	58.0	1.1	Green	yellowish
SPJ 53-21-24	153.5	122	9	21.25	200.5	84	23.25	16.65	0.75	0.88	35.0	1.1	41.0	1.1	Green	Light purple
SPJ 53-21-25	151.5	123	8	22.8	199	82	21.35	16.65	0.75	0.87	47.5	1.2	56.0	1.2	Green	Green
SPJ 53-21-50	177	125	10	24.95	203.5	86	23.05	16.85	0.75	0.87	35.0	1.2	62.0	1.2	Green	Light purple
SPJ 53-21-51	152.5	123	10	24.95	208.5	82	21.5	17.95	0.745	0.87	43.0	1.1	69.0	1.1	Green	yellowish
SPJ 53-21-52	177	121	11	21.9	200.5	83	23	16.65	0.75	0.85	34.0	1.0	43.0	1.0	Green	yellowish
SPJ 53-21-67	178.5	122	9	24.95	203	85.5	21.05	17	0.745	0.86	36.0	1.2	54.0	1.2	Green	yellowish
SPJ 53-21-71	176.5	120	10	25.6	208	85	20.1	16.85	0.74	0.875	46.0	1.1	63.0	1.1	Green	yellowish
SPJ 53-21-76	182	123	11	25.45	205.5	84	21.65	16.55	0.74	0.88	38.0	1.0	70.0	1.0	Green	yellowish
SPJ 53-21-77	183	124	12	23.4	203	86	19.5	18.2	0.74	0.89	39.0	1.2	54.0	1.2	Green	yellowish
SPJ 53-21-27	161.5	123	11	24.75	200.5	85.5	21.8	17.15	0.725	0.88	32.5	1.5	55.0	1.5	Green	yellowish
SPJ 53-21-15	158.5	122	9	26.05	196.5	83.5	20.1	18.3	0.74	0.86	33.6	1.4	47.0	1.4	Green	yellowish
SPJ 53-21-38	170	119	10	26.05	196.5	87	22.05	17.15	0.72	0.89	34.2	1.3	56.2	1.3	purple	Purple
SPJ 53-21-66	153.5	125	11	25.05	198.5	86	21.95	16.8	0.73	0.88	33.7	1.4	58.5	1.4	Green	yellowish
SPJ 53-21-13	169.5	124	11	25.15	204.5	88	21.9	18.25	0.74	0.87	34.1	1.5	51.7	1.5	Green	yellowish
SPJ 53-21-36	152	123	12	24	208.5	84	21.8	16.3	0.74	0.88	34.4	1.3	53.4	1.3	Purple	Purple
CRMAS 2232-85	105.5	105	12	24.15	218	84.5	18.2	20.5	0.72	0.84	34.0	2.0	33.0	2.0	Green	yellowish
Jalmagna	174	130	9	24.95	186	85.5	23.2	17.35	0.75	0.88	28.0	1.5	52.0	1.5	Purple	Purple
LSD_5%_	15.85		3.075	1.68	46.67	7.01	1.42	4.47	0.86	0.108	6.048	0.424	7.76	0.47		
CV%	4.7		14.1	3.3	11.0	4.0	3.2	12.5	5.6	6.0	7.7	16.2	6.7	17.8

### Background selection

The background selection was carried out for estimating the recurrent parent’s genome content in the pyramided lines. Background selection was performed by using 60 SSR markers among the lines possessing three resistance gene combinations in BC_1_F_1_, BC_2_F_1_ and BC_3_F_1_ generations. At BC_3_F_1_ generation, a total of 120 alleles from 60 markers were observed. The similarity co-efficiency among all lines ranged from 0.791 to 0.952 suggesting a high level of genetic similarity between the pyramids and Jalmagna. The dendrogram generated using the SSR data grouped the 14 three-gene pyramid lines into two major clusters (Figure 4B) with cluster I having CRMAS 2232–85 and rest 13 pyramided lines were clubbed in cluster II along with Jalmagna. Few members of cluster II *viz.,* SPJ53-21-77, SPJ53-21-25, SPJ53-21-52 and SPJ53-21-18 were very close to Jalmagna with 97, 95.33, 92.48 and 92.48% respectively.

### Analysis of genome introgression on the carrier and non-carrier chromosomes

**Figure 5:**
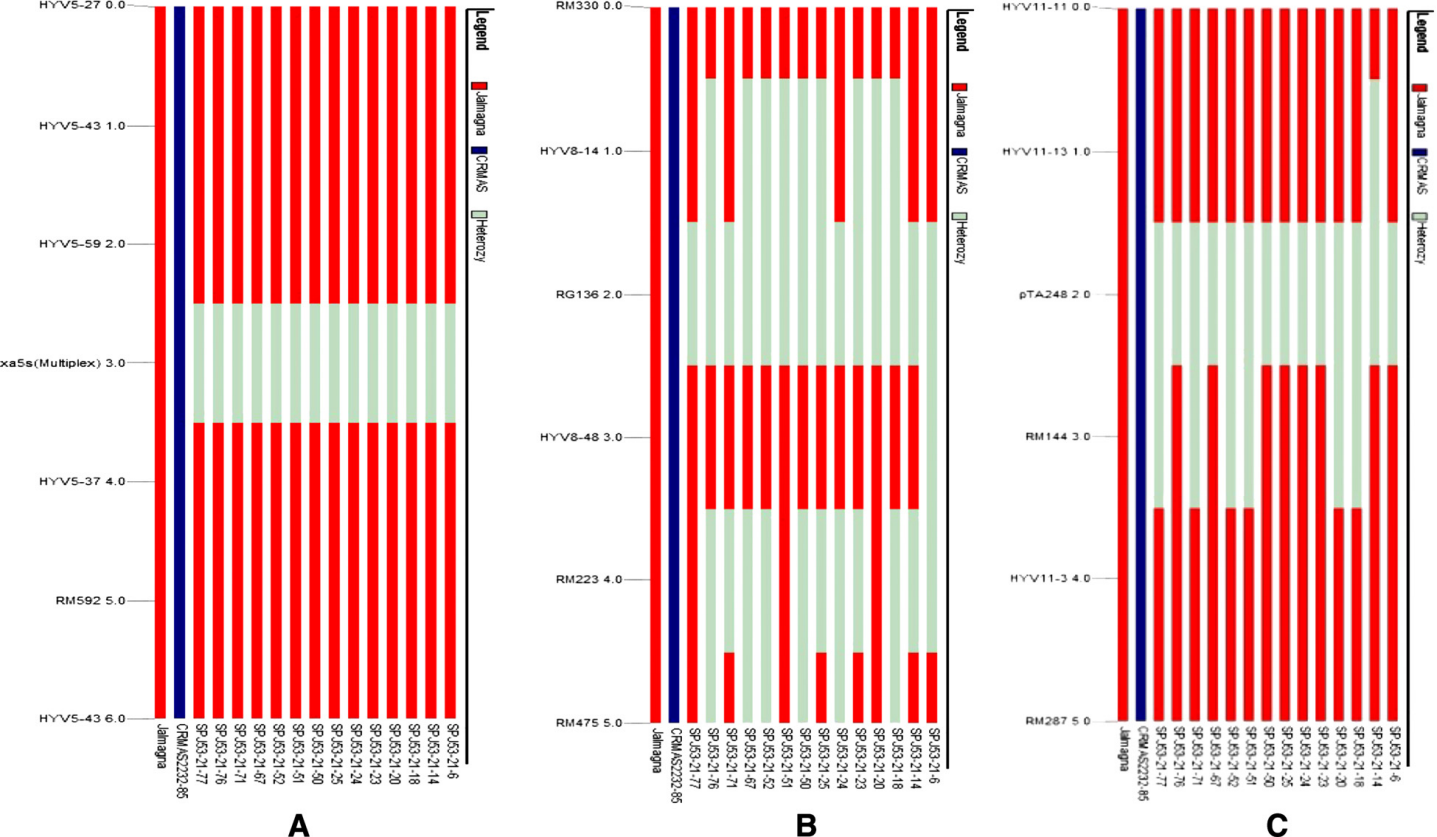
Analysis of genome introgression of 14 pyramided lines associated with resistance genes (**A**) *xa5* on chromosome 5; (**B**) *xa13* on chromosome 8 and (**C**) *Xa21* on chromosome 11 in Jalmagna and CR MAS 2232-85 BC_3_F_3_ derivatives.

In CRMAS2232-85/Jalmagna combination, 5–6 microsatellite markers on each of three carrier chromosomes in the genomic region flanking to *xa5*, *xa13* and *Xa21* were polymorphic. Based on six markers analysis, all the 14 lines showed heterozygosity for donor segment introgression of *xa5* between marker HYV59 and HYV5-37 in BC_3_F_1_ generation while exhibited homozygocity for Jalmagna genome and no drag to *xa5* gene was observed. In the flanking region of *xa13*, for five polymorphic markers, nine lines showed introgression of the donor segment of marker HYV14. In case of *Xa21*, six pyramid lines showed genetic drag of donor segment with the marker segment RM144 (Figure 5).

## Discussion

Integration of molecular markers to the backcross breeding was highly effective for transfer of three bacterial blight resistance genes. Phenotypic selections in three backcrossing and two selfing generations coupled with SSR based background selection was sufficient for transfer of *Xa21*, *xa13* and *xa5* genes into popular deepwater variety Jalmagna background. Jalmagna is a very long duration and strongly photo-sensitive variety. Again under deepwater situation, control of the disease using chemicals was a very difficult task. Here, integrating molecular markers and using very less generations and advancement of some generations under RGA helped to obtain the broad spectrum BB resistant lines for deepwater ecology. The transferred genes in the recombinants did exhibit high level of resistance against the most virulent BB isolates that is comparable to the reaction level of CRMAS 2232–85, the donor parent and the results are similar to earlier reports (Huang et al. 1997; Sanchez et al. 2000; Singh et al. 2001; Shanti et al. 2001; Bharatkumar et al. 2008; Hu et al. 2008; Perez et al. 2008; Sundaram et al., 2008; Rajpurohit et al., 2011; Dokku et al., 2013; Suh et al., 2013). The three gene combination pyramided lines expressed higher levels of resistance in comparison to parental lines, two and single gene combination. The results suggest that two gene combinations with *Xa21* + *xa13* was most effective with shorter lesions lengths followed by *Xa21* + *xa5* while lines with xa*13* + *xa5* were relatively less effective. Lines with *Xa21* in combination with either *xa5*, *xa13*, or both have shown promise advocating the utility of *Xa21* in achieving higher levels of resistance in rice as reported earlier (Singh et al. 2001; Sanchez et al. 2000; Sridhar et al. 1999; Huang et al. 1997) suggesting that synergistic action and/or quantitative complementation between the resistant genes might result in enhanced levels of resistance (Sanchez et al. 2000).

All the three resistance genes that have been considered in the present work have been cloned and characterized. *Xa21* is a dominant resistance gene that encodes a receptor kinase containing NBS-LRR domains (Song et al. 1995), while *xa5* is a recessive resistance gene and encodes a variant form of transcription factor cIIa (Iyer and McCouch 2004). The *xa13* resistance gene is also recessive in nature and has been shown to be a mutation in the promoter region of a gene that is a homolog of the nodulin MtN3 (Chu et al. 2006b). In rice lines containing the dominant (susceptibility) allele of the gene, the expression of the nodulin homolog is up regulated upon infection with *Xoo*. It appears that the increased expression of this gene is necessary for *Xoo* to grow on rice. This up regulation does not occur in rice lines containing the resistance (recessive) *xa13* allele (Yang, 2006). The apparently different modes of action of the three resistance genes used in this work might contribute to make the resistance in the three-gene pyramid lines quite durable. There is a variation in the theoretically expected value of contribution from the recurrent parent genome to the BC_1_F_1_ plants and in other backcross generations. As per reports of Sundaram et al. 2008, there might be exercising a “pull” for introgression of the *Xa21*, *xa13* and *xa5* genes during selection, which favors inheritance of additional unlinked loci from the donor genome in BC_1_F_1_ plants and BC_2_F_1_ generation. But, we found no pull effect during the transfer of *Xa21*, *xa13* and *xa5* genes to different backcross generations.

Selection of plants similar to the recurrent parent from BC_3_F_1_ stage was the strategy followed in the study and the graphical genotyping data supports that view as genotype SPJ53-21-77 had 97% of the recurrent parent genome having donor segments of target resistance genes *xa5*, *xa13* and *Xa21* and further no linkage drag in regions flanking *Xa21*, *xa13* and *xa5* is observed. The high recurrent genome recovery observed in many pyramid lines may be due to the use of more number of polymorphic microsatellite markers. Similar results were obtained in case of Sundaram et al. 2008; Dokku et al. 2013; Suh et al. 2013 suggesting more number of background markers. No genetic linkage drag was observed for the transfer of genes *Xa21*, *xa13* and *xa5* (Figure 5) may be due to mega variety used as the donor source for BB resistance genes. The mega variety, Swarna is a highly adapted variety for the favorable ecology. Results indicated that a broad based highly adapted variety as source of donor may give better performance and less drag as compared to the wild and land races as donor. It is expected that all the favorable genes are accumulated in the mega variety and subsequently transfer of some of these genes is improving further the background of the pyramided lines. The approach used in the study ensured the realization of the major objective resulting in the release of a cultivar with enhanced resistance to BB and accelerated recovery of recurrent genome with better yield.

Yield and agro-morphologic data of 20 pyramided two parental lines revealed that the pyramided lines possessed excellent features of recurrent parent and also yielding ability with tolerance to bacterial blight resistance. This indicates that some pyramid lines are very close to the recurrent parent and some are even better than the recurrent parent with respect to yield. The higher yield of the pyramided lines may be due to inheritance of some yield traits or QTLs of mega variety (Swarna BB pyramid) used here as the donor parent, besides the recurrent parent Jalmagna to the pyramided line. The complete recovery of the yield and grain quality characters of Jalmagna along with transfer of three BB resistance genes of CRMAS 2232–85 is a very significant achievement. This is particularly so because yield and agro-morphologic traits is multigenic traits encoded by loci that are distributed across the rice genome. The traits recovery of Jalmagna was due to integration of many polymorphic markers in the backcross breeding program. As per our analysis, we find that there is a variation from the theoretically expected 75% contribution from the recurrent parent genome to the BC_1_F_1_ plants. All the selected BC_1_F_1_ plants had a recurrent parent genome contribution more than the expected 75%. Again in BC_2_F_2_, it had highest gemone content of 91.8% along with the target genes. During BC_3_F_1_, the genome content of Jalmagna in selected derivative (SPJ53-21-77) was as high as 97%. The background selection with many markers accelerated the recovery of recurrent genome suggests that selection for introgression of the *Xa21*, *xa13* and *xa5* genes has no antagonistic effects for yield and other traits.

The field evaluation of BC_3_F_3_ progenies showed that the best entry had better yielding than the Jalmagna parent. Besides, BB resistance, the pyramided line. SPJ53-21-77 was better yielder than its recurrent parent and equivalent to agro-morphological traits and grain quality features of the recurrent parent. The high levels of resistance to BB and the absence of any yield penalty due to accumulation of resistance genes in the pyramids provides us a successful example of the integrated approach of selection at both molecular and phenotypic levels for transfer of the desired trait(s) and recovery of the recurrent parental genome. Development of broad-spectrum resistance against BB in the Indian subcontinent is a major challenge due to the rich diversity of the agro-climatic zones where rice is cultivated, as well as the presence of a number of genetically distinct virulent *Xoo* strains in different geographical areas of India. Deployment of a three gene combination like *xa5* + *xa13* + *Xa21* can achieve durable and broad-spectrum resistance in many BB prone rice growing areas in India including the deepwater ecosystem. The study clearly establishes the utility of MAS in pyramiding recessive genes like *xa5* and *xa13*, and dominant gene *Xa21* to present a multiple gene barrier against one of the most destructive diseases of rice in a long duration, photosensitive and deepwater rice.

## Conclusion

Marker-assisted backcrossing using functional markers reduce the risk of false selection in recombination between the molecular marker and the gene of interest. We were successful in identifying superior recombinations for three BB resistance genes (*Xa21, xa13 and xa5*) in the homozygous condition in a long duration, photosensitive and deepwater rice variety. The pyramided genotypes can be further be used for multi-location testing to be released as variety in the country or be used as potential BB resistance donors. The BB pyramided deepwater breeding lines, which are developed through MAS and phenotypic selection, will be of practical value in providing durable bacterial blight resistance in the deepwater growing region where control through chemicals under deepwater situation was less effective. These BB pyramided lines are expected to have a high impact on the yield stability and sustainability of deep water rice production.

## Methods

### Plant materials and breeding method

**Figure 6:**
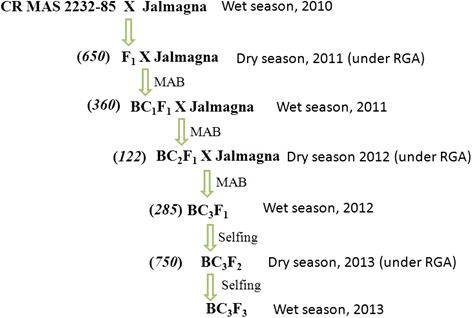
Schematic diagram for Pyramiding bacterial blight resistance genes into variety, Jalmagna through MAS (Figures in parentheses indicate the number of hybrids/ lines raised in that generation).

The donor parent CRMAS 2232–85, a derivative of Swarna and IRBB 60 cross contains three BB resistance genes *xa5*, *xa13* and *Xa21* in the background of mega variety Swarna. The donor parent was developed at Central Rice Research Institute (CRRI), Cuttack, India (Sundaram et al. 2014). The recurrent parent was Jalmagna, a highly popular variety of deepwater ecosystem of India but highly susceptible to bacterial blight disease. Jalmagna was hybridized with CRMAS 2232–85 and F_1_ plants were backcrossed with recipient parent Jalmagna. Marker-assisted backcross method was followed up to BC_3_ generation and around 200 plants/lines were genotyped at each generation for the presence of the target genes and only positive plants having the resistance alleles were advanced to the next generation. Foreground selection continued till BC_3_F_3_ to identify pure homozygous lines for all three target genes while background selection was up to BC_3_F_1_ generation. Selection based on foreground, background and morphological traits was practiced from BC_1_F_1_ onwards for identification of lines that were similar to the recurrent parent. Rapid Generation Advancement (RGA) facility was used during dry season as Jalmagna was a strongly photo-sensitive and very long duration variety. The schematic diagram for development of BB pyramided lines is presented in Figure 6.

### Screening for bacterial blight resistance

For field evaluation against BB, the inoculums of eight predominant *Xoo* isolates of Orissa prepared by suspending the bacterial mass in sterile water to a concentration of aproximately10^9^ cells/ml (Kauffman et al. 1973). Four leaves from four different plants of each entry were clip inoculated at the maximum tillering stage and lesion lengths (LL) were recorded after 15 days. The disease symptoms were scored as resistant (R, LL ≤ 3.0 cm), moderately resistant (MR, 3.0 cm < LL ≤ 6.0 cm), moderately susceptible (MS, 6.0 cm < LL ≤ 9.0 cm) or susceptible (S, LL > 9.0 cm) (Amante-Bordeos et al. 1992).

### Characterization for agro-morphological traits

Thirty days’ old seedlings of the BC_3_F_3_ pyramid lines and the parents (Jalmagna and CRMAS 2232–85) were transplanted in three rows with twenty five plants per row per entry at 15 × 20 cm spacing under a randomized complete block design with two replications at the experimental farm of Central Rice Research Institute (CRRI), Cuttack. Data were recorded on ten plants from each entry and replication for agronomic traits like plant height, tillers/plant, panicle length, number of filled grains/panicle, 1000-grain weight, flag leaf, 2^nd^ leaf length and breadth while days to 50% flowering was recorded on whole plot basis data analysis was performed using SAS statistical software (SAS Institute Inc. 2010).

### DNA isolation and PCR amplification

Mini scale DNA isolation for PCR analysis was carried out as per Dellaporta et al. (1983). The PCR reaction mixture contained 50 ng templates DNA, 5 pico mole of each of the primers, 200 μM dNTPs, 1 X PCR buffer (10 mM Tris–HCl, pH 8.3, 50 mM KCl, 1.5 mM MgCl2, and 0.01 mg/ml gelatin) and 0.6 unit of Taq DNA polymerase in a volume of 20 μl and amplification of target sequences were as per earlier reports (Table 1). The PCR products of STS marker RG 136 were digested with restriction enzymes *Hinf*I as per manufacturer’s instructions. The PCR products and the DNA fragments produced by restriction digestions were separated by gel electrophoresis and gel images were analyzed on gel documentation system (SynGene).

### Marker analysis

The primers employed for the three target genes were all from published reports (Table 1). Of the 236 SSRs markers used for parental polymorphism survey, 120 were found to be polymorphic between the parents (range 4–6 per chromosome) and 60 were used for background selection. Data were analyzed and similarity matrix was constructed from binary data with Jaccard’s coefficients and dendrogram was generated with unweighted pair group method arithmatic average (UPGMA) algorithm, using FreeTree software (Hampl et al. 2001; Pavalíce et al. 1999) and the dendrograms were visualized by Treeview 32 software (Page 1996). Graphical Geno Types (GGT) Version 2.0 (Van Berloo 1999) software programme was used for the assessment of the genomic contribution of the parent in the selected recombinants based on SSR marker data.
